# Exploring the Balance Between Artificial Intelligence and Human Expertise in Shaping Breast Reconstruction Outcomes: A Comparative Reflection Study

**DOI:** 10.3390/jcm15031170

**Published:** 2026-02-02

**Authors:** Ioan Constantin Pop, Maximilian Vlad Muntean, Vlad Alexandru Gata, Radu Alexandru Ilies, Delia Nicoara, Claudiu Ioan Filip, Vasile Pop, Patriciu Andrei Achimas-Cadariu

**Affiliations:** 1 Faculty of Medicine, “Iuliu Hațieganu” University of Medicine and Pharmacy, 400012 Cluj-Napoca, Romania; drp.ionut@gmail.com (I.C.P.); ilies.radu.alexandru@elearn.umfcluj.ro (R.A.I.); drdelianicoara@gmail.com (D.N.); 2 Department of Plastic Surgery, “Prof. Dr. I. Chiricuță” Institute of Oncology, 400015 Cluj-Napoca, Romania; 3 Department of Plastic and Reconstructive Surgery, “Iuliu Hațieganu” University of Medicine and Pharmacy, 400012 Cluj-Napoca, Romania; 4 Department of Surgical Oncology and Gynecologic Oncology, “Iuliu Hațieganu” University of Medicine and Pharmacy, 400012 Cluj-Napoca, Romania; pachimas@umfcluj.ro; 5 Department of Surgical Oncology, “Prof. Dr. I. Chiricuță” Institute of Oncology, 400015 Cluj-Napoca, Romania; 6 Department of Quality Management, “Prof. Dr. I. Chiricuță” Institute of Oncology, 400015 Cluj-Napoca, Romania; 7 First Surgical Clinic, Faculty of Medicine, “Iuliu Hațieganu” University of Medicine and Pharmacy, 400012 Cluj-Napoca, Romania; filip.claudiu.ioan@elearn.umfcluj.ro; 8 Department of Plastic Surgery and Burn Unit, Emergency District Hospital, 400535 Cluj-Napoca, Romania; 9 Department of Oral and Maxillofacial Surgery, Bihor County Emergency Hospital, 410169 Oradea, Romania; dr.popvasile13@gmail.com

**Keywords:** breast reconstruction, artificial intelligence, ChatGPT, patient education, oncological surgery, postoperative care

## Abstract

**Background/Objectives**: Artificial intelligence (AI) has shown potential in patient education and integration into clinical decision support systems. However, its performance in counseling patients on breast reconstruction currently remains underexplored. This study’s objective is to compare AI-generated answers with expert surgeon responses to common patient questions (derived from clinical scenarios) in domains like oncological justification, reconstructive options, and postoperative care. **Methods**: We realized an observer-blinded study using five real-world clinical scenarios in the field of oncologic and reconstructive surgery of the breast. Both ChatGPT-5 (October 2025 version) and a senior board-certified plastic surgeon responded to frequently asked questions, which were split into three domains: (1) oncological and surgical justification; (2) reconstruction options and outcomes, respectively; and (3) postoperative period. The answers were evaluated by another senior plastic surgeon using a four-grade ordinal scoring system (1 = unsatisfactory, 4 = excellent), which assessed accuracy, completeness, safety, nuance, and alignment with the current guidelines. **Results**: Across a total of 40 questions, the average AI response score was 3.1 ± 0.6. Domain-specific items scored lowest values for oncological justification (2.8 ± 0.7) and higher values for reconstruction options/outcomes and postoperative care (both 3.2 ± 0.4). No AI response was graded as unsatisfactory (score 1). Responses graded 4 (15%) were considered comprehensive, accurate, and patient-friendly. **Conclusions**: Globally, ChatGPT-5 provides satisfactory, readable, and medically accurate answers to basic patient questions on breast reconstruction, with a few limitations in nuanced oncological justification.

## 1. Introduction

According to the American Cancer Society, the incidence of breast cancer in women continues to rise, especially in young women under 50, with an annual increase of 1.4% over the last 10 years. Due to advances in treatment and early detection, mortality caused by breast cancer decreased in the last 30 years by 44% [1,2]. The options for surgical treatments of breast cancer are divided into two options: mastectomy and breast conserving surgery with whole breast radiotherapy. Although both options have equivalent survival rates, the total number of mastectomies has increased in recent years, with 35–40% of women diagnosed with breast cancer opting for this treatment. Some women also decide on contralateral prophylactic mastectomy even without a genetic predisposition or oncology recommendation [3,4].

Breast reconstruction and oncoplastic surgery offer patients with breast cancer a wide variety of advantages, including aid in recovery and a relieved adaptation after cancer treatment, enhanced body image and symmetry, and increased quality of life. Despite these advancements and the fact that in many countries the costs of reconstruction are covered, less than one in four women undergo immediate breast reconstruction after mastectomy. Such values highlight the need to improve access and educate patients about the available options [4].

Recently, artificial intelligence (AI) has become a popular field derived from computer science. Its function is to generate systems that involve tasks requiring human intelligence. The complexity of AI algorithms is high, involving features like pattern recognition, decision-making, and, of course, problem-solving. Domains, including machine learning, deep learning, and radiomics, are subsets of AI. Using them, AI models can further extract data from radiological images, having significant potential to transform and optimize medical specialties like Radiology and Imaging [5].

A new era started when a large language model (LLM) chatbot named Chat Generative Pre-trained Transformer (ChatGPT) was launched (30 November 2022), as it demonstrated various capacities, with the following examples: explanation of complex concepts, contribution by writing scientific literature, or essays with numerous topics. What is more, other complex processes, such as teaching, coding, marketing, translating tasks, summarizing movies, books, etc., were transformed by the use of artificial intelligence. Medicine has also undergone changes, as ChatGPT has shown consistency in various tasks: aiding the decision-making process in Radiology, providing a list of differential diagnoses, and, eventually, a final diagnosis [6].

The potential applications of ChatGPT in the medical and scientific communities are various. However, several features, such as its efficiency in performing literature searches in a comprehensive manner, data analysis, and even report writing on topics about plastic surgery, still remain unknown. Some problems regarding AI in supporting medical tasks, delivering responses are the lack of depth, delivering non-existent citations, and providing a superficial overview of the subject [6].

In the field of Plastic and Reconstructive Surgery, ChatGPT could potentially provide basic and general information in a coherent way and an accessible format on numerous topics, with examples of rhinoplasty and breast augmentation. ChatGPT is believed to serve as a highly valuable resource with the aim of supplementing, and not replacing, humans. Its interaction with medical specialists (who can offer more specific and personalized information) can be beneficial for the patient. In order to improve the integration of AI chatbots in medicine, efforts ought to be made to enhance their empathy, allowing for individualized interaction with every patient [7].

AI is responsible for transforming reconstructive breast surgery by optimizing a wide variety of processes like preoperative planning, intraoperative precision, postoperative management, and even academic training. Preoperatively, AI turned out to improve risk prediction and patient counseling. Intraoperatively, it aids in identifying the anatomical landmarks, making use of the latest technologies designed for 3D visualization. In addition to this, assistance for complex procedures can be obtained using AI, applied for performing microsurgical anastomosis. Postoperatively, artificial intelligence is useful for optimizing follow-up care and resource allocation. Academically, it provides physicians with personalized training and supports research. Even though AI appears to show an immense potential in breast reconstruction, various challenges such as privacy concerns, high costs, and operational efficacy still remain [8].

Apart from technical feasibility, breast reconstruction implies complex and individualized patient counseling, integrating oncologic safety, patient preferences, reconstructive options, and postoperative expectations. This process is very nuanced and is traditionally based on the surgeon’s clinical experience, communication skills, and the ability to adapt information to each patient’s oncologic and psychosocial context. In this setting, the potential role of large language models as supportive tools in patient counseling remains insufficiently explored.

The current study aims to evaluate the answers that are generated by ChatGPT-5, in comparison to the views of the plastic surgeon regarding the planning of breast reconstruction. Furthermore, the capacity of AI to properly guide therapy in plastic surgery (in this case, breast reconstruction) is not only assessed but also questioned. This study focuses on emphasizing both advantages and disadvantages of using AI models in this case, respectively, to predict their evolution in the future, as the integration of AI in medicine is currently debated.

## 2. Materials and Methods

We performed an observer-blinded, cross-sectional study comparing AI-generated and expert responses to breast reconstruction–related questions on the following topics: oncological and surgical justification; reconstruction options and outcomes; and postoperative care.

In order to ensure a standardized comparison between AI-generated and surgeons’ responses, we described five clinical scenarios consisting of common patient presentations in the field of breast oncological surgery and breast reconstruction. Each patient case was similar to a real-world clinical encounter, reflecting different oncologic stages, therapeutic pathways, reconstructive indications, and patient concerns.

We asked ChatGPT (OpenAI, San Francisco, CA, USA, GPT-4o model, October 2025 version, which is the most recent version of this program) some frequent questions about breast reconstruction by creating scenarios. The same questions were answered by a plastic surgeon from our university center, specialized in breast reconstruction, and then the responses were evaluated by another plastic surgeon with experience in the same field (who was blinded to the identity of the respondent). Our first step was to identify the most frequent questions about breast reconstruction on websites on this topic, which are dedicated to patients, and then to select the most relevant questions. Hence, a standardized comparison between AI-generated and expert-derived responses was possible.

The questions were classified into three groups based on the following themes:

1. Oncological and surgical justification.
2. Reconstruction options and outcomes.
3. Postoperative period.

### 2.1. Evaluation Scale

The quality of the responses was assessed using a four-point ordinal scoring system grounded in objective criteria, including completeness, accuracy, informational safety, nuance, and alignment with current clinical guidelines in oncologic breast surgery and reconstruction. The detailed score based on the criteria mentioned is provided below:

- Score 4—Excellent response requiring no clarification:
  A comprehensive, well-structured, and scientifically accurate answer, clearly formulated, free of potentially misleading statements, offering sufficient nuance and fully aligned with established clinical recommendations.
- Score 3—Satisfactory response requiring minimal clarification:
  A generally correct and adequately informative response, with minor omissions or areas that could benefit from slight elaboration, without posing a meaningful risk of misinterpretation.
- Score 2—Satisfactory response requiring moderate clarification:
  A partially complete or somewhat ambiguous answer, containing gaps or imprecisions that may affect interpretation; additional corrections are required to ensure clarity and prevent patient misguidance.
- Score 1—Unsatisfactory response requiring substantial clarification:
  An incomplete, inaccurate, or insufficient response with a potential risk of misleading the patient or compromising clinical decision-making, lacking alignment with current best practice guidance.

In order to evaluate the readability of the responses, the Flesch–Kincaid Grade Level score was used, making it possible to determine the educational level that is required to understand the text.

This study did not involve any human participants, patient data, or identifiable personal information. Because all materials were derived from publicly available educational content and simulated clinical scenarios, the current study qualified for exemption from institutional ethical review.

### 2.2. Patient Scenarios and Key Clinical Questions

**Scenario 1**

A 56-year-old female presents with right breast cancer, T4bN2aM0. Right mastectomy and right axillary lymphadenectomy are recommended. After researching various specialized websites, the following questions were selected:

1. Why is a sectorial resection not possible, and why was mastectomy recommended instead?
2. Why is immediate reconstruction not feasible?
3. When can breast reconstruction be performed?
4. What are the options for breast reconstruction?
5. What are the differences between the two reconstruction options: implant-based vs. autologous tissue?

**Scenario 2**

A 48-year-old female presents with right breast cancer, T2aN0M0. Right mastectomy and sentinel lymph node biopsy of the right axilla are decided, along with pre-pectoral implant-based breast reconstruction.

1. Why is sectorial resection not performed?
2. What is a sentinel lymph node biopsy? Why is lymphadenectomy not performed?
3. Are there other options for breast reconstruction?
4. Does breast reconstruction impact subsequent monitoring and follow-up?
5. Will the implants need to be replaced after a few years?
6. What are the risks associated with immediate reconstruction using prostheses?
7. What impact will the surgery have on sensation in the breast?
8. Do I have to wear a medical compressive bra postoperatively? How long?
9. When can I resume household activities?
10. How can I avoid complications?

**Scenario 3**

A 50-year-old female presents for breast reconstruction following previous right breast cancer surgery and chemoradiotherapy 5 years earlier. She is recommended for tissue expansion for breast reconstruction due to a lack of donor tissue for autologous reconstruction.

1. What is a tissue expander, and why cannot direct reconstruction with an implant be performed?
2. After how long will tissue expansion begin, and what is the typical timeline for this process?
3. When will the tissue expander be replaced?
4. How long can the expander be kept in place?
5. How can symmetry be achieved in breast surgery, considering the other breast has a degree of ptosis?
6. When can I drive the car after the surgery?
7. How should I take care of the expander?

**Scenario 4**

A 42-year-old female presents for breast reconstruction 8 years after undergoing left breast cancer surgery and chemoradiotherapy. She is recommended for autologous tissue breast reconstruction (DIEP flap).

1. What are the benefits of this procedure compared to breast implants?
2. What are the associated risks?
3. How long will I stay in the hospital?
4. What is the recovery period postoperatively?
5. Can breast symmetry be achieved during the same surgery?
6. When can I return to work?
7. When will I be discharged?
8. When will I be completely recovered?
9. What are the signs of complications?

**Scenario 5**

A 35-year-old female presents with left breast cancer, T1cN0M0. Oncoplastic sectorial resection and sentinel lymph node biopsy are recommended.

1. Is this procedure as oncologically safe as a mastectomy?
2. What are the benefits compared to mastectomy and immediate breast reconstruction?
3. Can breast symmetry be achieved during the same surgery?
4. How will the breast look postoperatively?
5. Will it affect future oncological follow-up?
6. Is any additional treatment required?
7. Will I be able to breastfeed after the surgery?
8. Will I have postoperative pain? How can I manage it?
9. Will there be visible scars? What can I do to make it look better?

## 3. Results

**Figure 1:**
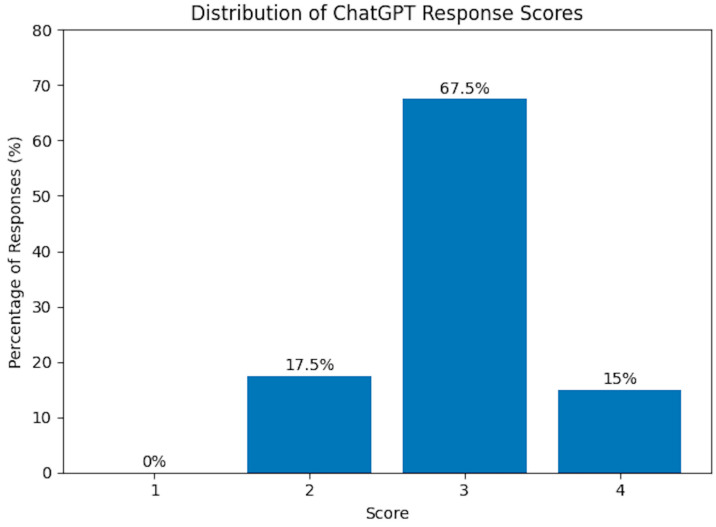
Distribution of scores assigned to ChatGPT responses.

Across all mentioned domains, the average score for ChatGPT responses was 3.1 ± 0.6, which corresponds to a generally satisfactory quality that only requires minimal clarification. None of the responses was graded as 1 (unsatisfactory). Out of the total, 7 responses (17.5%) were rated 2, 27 responses (67.5%) received a score of 3, and the remaining 6 responses (15%) achieved a perfect score of 4 (Figure 1).

The comparison between the scores of the responses delivered by the physician and the AI model is summarized in Table 1.

### 3.1. Domain 1—Oncological and Surgical Justification

This section was composed of eight questions addressing oncological explanations and surgical indications. The average score for this domain was 2.8 ± 0.7, representing the lowest performance among the three categories.

Responses graded as 2 were noticed for questions that aimed to address nuanced oncological reasoning, including the following:

- “Why is a sectoral resection not possible, and why was mastectomy recommended instead?”
- “What is a sentinel lymph node biopsy? Why is lymphadenectomy not performed?”
- “Does breast reconstruction impact subsequent monitoring and follow-up?”
- “How can symmetry be achieved in breast surgery, considering the other breast has a degree of ptosis?”

The highest rating (4) was constantly attributed to concise and clear explanations (e.g., “Is this procedure as oncologically safe as a mastectomy?”).

### 3.2. Domain 2—Reconstruction Options and Outcomes

This domain was composed of 13 questions about reconstructive timing, surgical techniques, and esthetic outcomes. The average score was 3.2 ± 0.4, corresponding to generally clear and informative answers.

The majority of responses represented accurate overviews of reconstruction methods and timing but were sometimes lacking in technical specificity, as reflected in the following questions:

- “Why is immediate reconstruction not feasible?”
- “What are the differences between the two reconstruction options: implant-based vs. autologous tissue?”
- “How can symmetry be achieved in breast surgery, considering the other breast has a degree of ptosis?”

Responses that were graded 4 (e.g., “What are the options for breast reconstruction?”, “What is a tissue expander, and why cannot direct reconstruction with an implant be performed?”) were characterized by clarity, completeness, and the use of accessible terminology suitable for patient education.

### 3.3. Domain 3—Postoperative Period

This domain was composed of 13 questions about reconstructive timing, surgical techniques, and esthetic outcomes. The average score was 3.2 ± 0.4, corresponding to generally clear and informative answers.

The majority of responses represented accurate overviews of reconstruction methods and timing but were sometimes lacking in technical specificity, as reflected in the following questions:

- “Why is immediate reconstruction not feasible?”
- “What are the differences between the two reconstruction options: implant-based vs. autologous tissue?”
- “How can symmetry be achieved in breast surgery, considering the other breast has a degree of ptosis?”

These answers were considered comprehensive, medically accurate, and reassuring.

### 3.4. Readability Analysis

The average value of the Flesch–Kincaid Grade Level across all AI-generated responses was 8.9 ± 0.7, attributed to an educational level that is equivalent to that of a high school graduate. This shows that the majority of patients who have average health literacy would be able to understand most of the provided explanations without significant difficulty.

### 3.5. General Overview of Results

Altogether, the performance patterns across different domains show that lower scores were associated with the questions that require oncologic justification and individualized clinical decision-making. Conversely, questions that were related to reconstruction options and postoperative care were linked to more uniform and higher scores, supporting the strengths of large language models in standardized informational counseling.

## 4. Discussion

The purpose of this section is to interpret the evaluated performance patterns and to contextualize them within key themes relevant to clinical practice, including adherence to clinical guidelines, empathy in patient counseling, and the current limitations of artificial intelligence.

### 4.1. The Role of AI in Patient Education and Surgical Support

In most cases, patients may not receive all the desired information from doctors, and they will probably search for their answer by making use of online forums, social media groups, or other online resources. Many patients might feel anxious during the consultation and may encounter difficulties processing or remembering information. On the other hand, there are some patients who can feel intimidated or hesitate to disturb the doctor. The limited consultation time and medical complex language can be responsible for gaps in information. Patients are often tempted to read about experiences like their own, which can lead them to seek information using unreliable or unverified sources that can sometimes be dangerous.

AI applications may offer a potential solution to this problem provided that the algorithms are trained on data from verified sources. Nevertheless, limitations remain, particularly in handling contradictory information across sources and in synthesizing the most relevant information according to patient needs. Their reliability might be improved in the future, but such models should accompany medical advice and not substitute it, due to legal consequences and ethical considerations. The main character should also be the physician, and AI programs can adjunct medical communication and patient support.

Availability and accessibility are two of the most important advantages that AI can provide to patients. It can offer quick and accurate information from a vast database, and it can alleviate the anxiety of patients until the meeting with the surgeon. This platform can educate patients before the consultation. In this case, patients are familiarized with this new subject and can have a better understanding of the information that is provided by the doctor, and can ask rational questions. In this way, AI can help medical staff by reducing the workload, without consuming valuable time for physicians.

In a surgical specialty like plastic surgery, where the aesthetic results play a more vital role, AI programs can be integrated into the evaluation process of breast symmetry, which has been primarily subjective until now, based on the surgeon’s vision and the patient’s self-evaluation. A few inconsistencies may appear due to a lack of parametric methods or mathematical models that can process the geometry behind the surgical outcomes in a visual interpretation. Kenig et al. evaluated the outcomes using an AI model, which calculated the symmetry of the breasts for the group of patients who underwent reconstructive surgery of the breast, demonstrating a high correlation with human evaluation of the same results, but offering the advantages of using precise measurements. The model successfully detected 399 out of the total 405 landmarks (98.51%), with an average calculation time of 0.92 s, 16 times faster than humans (average of 14.09 s). It can enhance time management and optimize workflow with acceptable accuracy. Thus, AI may be beneficial in such cases, to be used for support and to give feedback for both the patient and the surgeon [9].

In addition to supporting patients and improving communication, a high variety of tasks can be performed by artificial intelligence, taking into account its predictive capacity, pattern recognition, extracting data, and optimizing decision-making. A systematic review conducted by Soh et al. assessed the application of machine learning in breast surgery, dividing its applications into four categories: predictive modeling, breast imaging-based context, screening and triaging of breast cancer patients, and network utility for detection. Machine learning turned out to outperform the traditional statistical methods in each analyzed study, in terms of predicting quality of life, mortality, and morbidity. It could aid planning, surgical navigation, and optimize the proper identification of anatomical structures [10].

### 4.2. Pros and Cons of AI in Patient Communication and Guidance in Plastic Surgery

AI can respond very fast and accurately to a patient’s answers about their problem, based on a very large database. Unfortunately, in some cases, patients may not understand their real problem and can ask ChatGPT wrong questions, which, in some cases, could be dangerous. AI cannot control the patient’s questions and sometimes can make wrong recommendations.

In medicine, empathy and the capacity to have a direct interaction with patients are essential. Doctors can adapt their explanations in every case and can give information to patients progressively. In this case, the patients can understand their problem and could better tolerate the unwanted, bad news. AI can emphatically offer information, but it cannot really understand the emotions of the patients.

Plastic surgeons are willing to offer only safe information with scientifically proven results. On the other hand, AI can have limitations in verifying the most recent information. When compared to data taken from breast reconstruction guidelines, the responses of AI lacked accuracy. A study conducted by Saturno et al. analyzed the concordance rate between ChatGPT and the American Society of Plastic Surgeons (ASPS) guidelines on several procedures in breast surgery, like Reduction mammaplasty, Breast reconstruction with expanders (or implants), and Autologous breast reconstruction with DIEP or pedicled TRAM abdominal flaps. Out of 10 questions about reduction mammaplasty, Chat GPT returned 5 fully concordant answers, 2 partially concordant, and 3 non-concordant. Furthermore, ChatGPT was asked about 17 topics related to breast reconstruction using prosthetic materials: 5 responses were fully concordant, 7 were partially concordant, and 5 were non-concordant. Finally, when it was asked about five topics on autologous breast reconstruction, four were partially concordant, and one was non-concordant. Its accuracy still requires improvements in order to be widely applied in the field of plastic surgery [11].

Although the information available online is of solid quality, patients seek confirmation from their treating physicians. When a patient decides to undergo treatment with a particular doctor, they develop a special trust in that doctor and form a close relationship. I believe that, although the information generated by artificial intelligence is accurate and valuable, it always requires confirmation from a doctor.

AI systems (like ChatGPT) usually fail to grasp the level of complexity in various surgical procedures, sometimes returning responses that are too simplistic and generalized. For instance, breast reconstruction using implants is substantially less complex than reconstruction procedures that are flap-based, for both the patient and the surgical team. Despite this, AI responses have a tendency to equalize these interventions in terms of difficulty, failing to emphasize the challenges (which are nuanced) associated with flap reconstruction, like extended operative time, higher rates of complications, and the need for microsurgical expertise (which requires a complex learning curve). This lack of distinction can lead to robotic and uniform answers that do not optimally inform patients about the reality of surgical options and their varying levels of complexity.

Even when AI-generated recommendations are in accordance with human surgeon responses, the reasoning process of the AI still remains largely opaque. For clinicians, it is vital to interpret and contextualize these outputs before making any decisions. Emphasizing this aspect optimizes the clinical relevance of AI-assisted patient counseling and reinforces the role of the human surgeon in ensuring safe care.

Recently, Retrieval-Augmented Generation (RAG) frameworks have been considered a method to optimize large language models by integrating external, curated knowledge sources. A systematic review conducted by Fnu Neha et al. explores RAG applications in diagnostic support, electronic health record summarization, and medical question answering. It identifies persistent challenges like retrieval noise, domain shift, latency, and limited explainability [12]. By incorporating RAG into clinical settings, AI has the potential to improve consistency, adherence to guidelines, and personalization in patient counseling.

### 4.3. Strengths and Limitations of the Current Study

By explicit comparison between AI-generated recommendations and human expert judgment, the current study underscores the complementary (rather than competitive) values of technology and surgical experience. It promotes a practical and balanced framework for AI integration in everyday clinical practice. Our study addresses a highly debated topic consisting of the intersection of artificial intelligence and reconstructive breast surgery, which represents an area with limited structured analysis. Thus, it responds directly to the growing interest in integrating AI-assisted clinical decision-making systems within personalized oncologic care.

Another strength of the study is the comparative approach of question-based tasks, derived from complex scenarios (such as immediate versus delayed reconstruction, or implant-based versus autologous techniques), which represents a valuable educational tool for residents and early-career specialists, emphasizing how AI can be used responsibly as a learning and support resource while illustrating the irreplaceable value of human expertise.

On the other hand, AI-generated recommendations with expert decision-making are fundamentally different processes. AI outputs depend on training data, design of the inserted prompt, and algorithmic constraints, whereas human expertise is mainly shaped by knowledge, combined with clinical intuition and real-time intraoperative judgment. This intrinsic heterogeneity strongly limits the equivalence between these two.

Additionally, the quality and clinical relevance of AI-generated outputs are mainly influenced by the accuracy, completeness, and structure of the input data. Variability regarding the formulation of the prompt might be a source of bias and further limit reproducibility across different settings or users.

Moreover, the present study focuses potential impacts of AI versus human expertise on breast reconstruction outcomes. It does not bring a direct causal relationship between AI-generated recommendations and actual postoperative results. Clinical outcomes remain primarily influenced by surgeon expertise and decisions of the multidisciplinary medical team.

It is important to mention that both AI systems and human physicians are subject to different sources of bias. AI models are influenced by training data, algorithmic architecture, and framing of the prompt, while the surgeons’ judgments are influenced by clinical experience, specialty-specific training, and cognitive processes. Clearly distinguishing algorithmic bias from human cognitive bias reinforces the conceptual basis of comparative evaluations and avoids implying that AI represents a neutral reference.

In addition, the evaluation of AI-generated responses was conducted by a single expert plastic surgeon. While this approach reflects real-world clinical judgment and expertise, it does not allow assessment of interobserver variability. Consequently, this might limit the reproducibility and generalizability of the findings, which should be further explored in future studies involving multiple evaluators with different levels of experience.

Given the rapid evolution of AI models and the continuous release of updated versions, the findings of this study may not fully generalize to future versions of AI systems. This temporal limitation should be taken into account when interpreting the results.

Globally, most of the limitations identified in the current study, including insufficient oncological nuance, lack of guideline grounding, and limited tumor-specific contextualization, could perhaps be addressed by RAG frameworks. Exploration of RAG-based systems represents an important avenue for future research.

It is important to note that the ChatGPT-5 setup, which was assessed in this study, represents a generic, non-retrieval large language model, being used without the integration of any external clinical guidelines or institution-specific protocols. Therefore, several observed deficiencies are caused by architectural constraints of the used model rather than inherent limitations of AI-assisted counseling in general.

### 4.4. Legal and Ethical Principles of AI Application in Medicine

The dependence of artificial intelligence on extensive datasets raises several legal concerns regarding data privacy and security. It is mandatory to manage every information that is available from patients in accordance with stringent ethical standards and strict regulations to ensure responsible usage. Robust data governance policies should be established to mitigate risks and protect patient confidentiality [13]. Furthermore, transparency in the process of decision-making performed by AI is crucial to foster trust between patients and healthcare providers. For being effectively incorporated into clinical workflows, AI-generated recommendations must be viewed and analyzed by medical professionals who have the responsibility to understand the mechanisms and limitations of the algorithms and to ensure their informed application in clinical practice. A collaborative approach between technologists and clinicians is essential to align AI innovations with ethical and practical healthcare needs [14,15,16].

The impact of AI on current healthcare brings several ethical considerations that require careful attention. Even if AI models may offer a significant potential to enhance diagnostic capacity and improve surgical outcomes, it is essential to view such technologies only as supportive for all healthcare professionals who use them, and not autonomous [15,16]. The process of making decisions is complex, and it demands a strong experience of specialists in their own field to integrate all the available data that is derived from the patients with other variables from imaging and laboratory analyses. Even more, the technological advances concerning AI should not overshadow the importance of human empathy and patient-centered care in medical practice [16,17,18,19].

## 5. Conclusions

ChatGPT showed a generally satisfactory performance in responding to patients’ questions about breast oncologic surgery and reconstruction, with particularly positive results in the postoperative and follow-up domains. While the majority of responses were clear, precise, and appropriate for patients who presented average health literacy, some limitations appeared in the areas that require complex oncological reasoning and detailed surgical justification. Thus, ChatGPT might represent a valuable and supportive tool for patient education and reassurance; however, its use must remain complementary to discussions with clinicians, especially when involving individualized oncological safety and surgical decisions. Future research could evaluate the integration of RAG frameworks to improve AI-assisted patient counseling in reconstructive surgery. While our study used a generic large language model, RAG-based systems can be the next step to provide more personalized and clinically grounded support for patients.

## Figures and Tables

**Table 1:** Assessment of clinical and AI responses to breast surgery questions.

Category	Question	Physician Response Score	AI Model Response Score
Oncological and Surgical Justification	Why is a sectorial resection not possible, and why was mastectomy recommended instead?	4	4
	2. Why is sectorial resection not performed?	2	2
	3. What is a sentinel lymph node biopsy? Why is lymphadenectomy not performed?	4	4
	4. Is this procedure as oncologically safe as a mastectomy?	3	3
	5. Does breast reconstruction impact subsequent monitoring and follow-up?	4	4
	6. Will it affect future oncological follow-up?	2	2
	7. Is any additional treatment required?	4	4
	8. Will I be able to breastfeed after surgery?	4	4
Reconstruction Options and Outcomes	Why is immediate reconstruction not feasible?	4	3
	2. When can breast reconstruction be performed?	4	3
	3. What are the options for breast reconstruction?	4	4
	4. What are the differences between the two reconstruction options: implant-based vs. autologous tissue?	4	3
	5. Are there other options for breast reconstruction?	4	3
	6. How can symmetry be achieved in breast surgery, considering that the other breast has a degree of ptosis?	4	2
	7. What are the benefits of this procedure compared to breast implants?	4	3
	8. Can breast symmetry be achieved during the same surgery?	4	3
	9. What are the associated risks?	4	4
	10. How will the breast look postoperatively?	4	3
	11. Will there be visible scars? What can I do to make them look better?	4	3
	12. What is a tissue expander, and why cannot direct reconstruction with an implant be performed?	4	4
Postoperative period	After how long will tissue expansion begin, and what is the typical timeline for this process?	4	4
	2. When will the tissue expander be replaced?	3	3
	3. How long can the expander be kept in place?	4	4
	4. When can I drive the car after the surgery?	3	3
	5. How should I take care of the expander?	4	4
	6. How long will I stay in the hospital?	3	3
	7. What is the recovery period postoperatively?	4	4
	8. When can I return to work?	3	3
	9. When will I be discharged?	4	4
	10. When will I be fully recovered?	3	3
	11. What are the signs of complications?	3	3
	12. Do I have to wear a medical compressive bra postoperatively? How long?	3	3
	13. When can I resume household activities?	4	4
	14. How can I avoid complications?	3	3
	15. Will the implants need to be replaced after a few years?	4	4
	16. What are the risks associated with immediate reconstruction using prostheses?	3	3
	17. What impact will the surgery have on sensation in the breast?	3	3
	18. Will I be able to breastfeed after the surgery?	3	3
	19. Will I have postoperative pain? How can I manage it?	3	3
	20. Is this procedure as oncologically safe as a mastectomy?	3	3

## Data Availability

Data are available upon reasonable request from the corresponding author.
